# Pharmacologic Treatments for the Preservation of Lean Body Mass During Weight Loss

**DOI:** 10.3390/jcm15020541

**Published:** 2026-01-09

**Authors:** Gunjan Arora, Katherine R. Conde, Cyrus V. Desouza

**Affiliations:** University of Nebraska Medical Center, Omaha, NE 68198, USA; garora@unmc.edu (G.A.); kaby@unmc.edu (K.R.C.)

**Keywords:** GLP-1 RA, GIP/GLP-1 RA, lean body mass loss, weight loss

## Abstract

**Introduction**: Overweight and obesity are becoming increasingly prevalent. Incretin-based obesity treatments—glucagon-like peptide-1 receptor agonists (GLP-1 RAs) and dual glucagon-like peptide-1 receptor/glucose-dependent insulinotropic polypeptide receptor agonists (GIP/GLP-1 RAs or dual agonists)—are a major stride in the evolution of obesity management. However, like weight loss with other means, they are associated with an inadvertent significant loss of lean body mass, including muscle. This has led to a resurgence in research for the preservation of lean body mass, the loss of which occurs with weight loss. The purpose of this narrative review is to discuss the mechanisms involved with lean body loss and capture the research landscape of the different classes of pharmacological agents being developed to address this problem. **Methodology**: We queried PubMed, Medline, and Scopus for randomized controlled trials and phase II or phase III trials using key words to capture the breath of this topic—obesity, weight loss, muscle loss, lean mass, and muscle preservation. Animal studies were excluded. We analyzed the studies conducted to date. **Results**: Weight loss, regardless of the method used to achieve it, is inadvertently accompanied by lean body mass loss, to varying degrees. There are several mechanisms that govern the loss of lean body mass and, more specifically, the loss of muscle mass; as such, several classes of medications have been explored, targeting different pathways and receptors—including bimagrumab (activin receptor agonist), tesamorelin (growth hormone releasing hormone agonists), and enobosarm (selective androgen receptor modulator). Most of these drugs are in the early phases of research development, but some show great promise. **Conclusion**: This narrative review attempts to detail the physiology of muscle mass loss when accompanied by weight loss and identify pharmacological targets that can be utilized to minimize it with mechanisms, effects, side effects, and research developmental progress.

## 1. Introduction

According to the Centers for Disease Control and Prevention (CDC), it is estimated that over 70% of adults in the United States (U.S.) are overweight or obese [1]. This translates to nearly 100 million adults in the U.S. alone. Thus, the need for treatment options for overweight and obesity is substantial. Recently, this demand has been partially met with the advent of incretin-based drug therapy—namely, the glucagon like peptide-1 receptor agonists (GLP-1 RAs) and the glucose-dependent insulinotropic polypeptide (GIP) receptor agonist in combination with the GLP-1 RA (GIP/GLP-1 RA or dual agonist) [2].

GLP-1 RAs and GIP/GLP-1 RAs were initially developed for the treatment of hyperglycemia in type 2 diabetes mellitus (T2DM) [2]. GLP-1 RAs and GIP/GLP-1 RAs mimic naturally occurring incretin hormones that promote satiety by delaying gastric emptying, reducing appetite-stimulating signals from various brain areas, including the hypothalamus (the hunger center), improving insulin secretion, and suppressing glucagon release [3]. Both GLP-1 and GIP have receptors in the brain and throughout the gastrointestinal tract, allowing them to modulate hunger and satiety through complex gut–brain axes, effectively reducing appetite and increasing satiety, in turn facilitating weight loss [3].

After showing significant benefit in controlling blood glucose levels and promoting weight loss in patients with T2DM, GLP-1 RAs and GIP/GLP-1RAs were approved by the Food and Drug Administration (FDA) for use in non-diabetic patients with overweight or obesity [4]. Currently liraglutide and semaglutide are the approved GLP-1 RAs and tirzepatide is the approved GIP/GLP-1 RA for weight loss; however, it is likely that we will see many more being approved in the near future [5]. The side effect profile for GLP-1 RAs and GIP/GLP-1 RAs consist mainly of mild-to-moderate nausea, vomiting, and diarrhea [6]. However, one important side effect is the loss of lean body mass, including muscle [7].

While the concern of lean body mass (LBM) or fat-free mass (FFM) loss with weight loss has been recently highlighted with the use of GLP-1 RAs and GIP/GLP-1 RAs, the fact remains that FFM loss is observed with all modalities of weight loss. The general rule for FFM loss with weight loss by any means is the “Quarter FFM Rule”, which states that a quarter of the total weight lost is attributable to loss of FFM [8]. While this rule is under scrutiny, studies on various weight loss modalities have shown that a variable percentage of total weight loss is attributed to the loss of FFM [8,9]. It is important to note that estimates of lean or fat-free mass can vary based on the methods used to achieve weight loss and measure them, and terms are often used interchangeably. When using MRI, DXA, or CT scans to estimate, FFM is the largest category, consisting of skeletal muscle, water, organs, and bone; LBM is skeletal muscle, organs, and water. Additionally, skeletal muscle mass alone can be measured and reported [10]. Even pure fat mass loss is associated with the loss of FFM when one considers the connective tissue that was lost which was providing support to the fat mass [8,9].

In a systematic review and meta-analysis of the effects of caloric restriction on muscle mass it was found that 25.5% of weight loss was due to muscle mass in those without diabetes, and in those with T2DM, weight loss due to muscle mass loss was 27.5% of the total weight loss [11]. Similar results have been shown many times over in studies looking at the effect of weight loss via caloric restriction on lean body mass. When looking at weight loss via bariatric surgery, a systematic review and meta-analysis showed that one year following surgery, 23.2% of weight loss was due to FFM [10]. In general, studies estimate that bariatric surgery yields 25–35% total weight loss with 25–30% of that being attributed to FFM loss [9]. These studies clearly show that FFM loss by more traditional mechanisms does indeed approach a quarter of total weight lost.

Among the GLP-1 and GIP/GLP-1 RAs that have received FDA approval for the treatment of obesity, tirzepatide showed the largest percentage of total weight lost, 20.9% during the 72-week SURMOUNT-1 trial with 24%, or just short of a quarter, of this weight being attributed to FFM loss [9,12]. Semaglutide showed a 14.9% total weight loss over 68 weeks in the STEP-1 trial, with 39% being attributed to FFM loss [9,13]. Finally, liraglutide showed less total weight loss of just 8.4% over 56 weeks; however, FFM loss was not evaluated [9,14]. While the amount of FFM lost using the newer pharmacologic methods is generally similar to that seen with caloric restriction and bariatric surgery, the number of individuals losing weight with pharmacologic methods has increased. Thus, preserving FFM while promoting fat loss is of great interest in treating overweight and obesity with favorable long-term effects of preserved muscle mass, such as slowing or preventing weight regain and improving metabolic parameters.

The detrimental effects of the loss of LBM in the setting of weight loss are widely recognized. As the number of patients using the GLP-1 and GIP/GLP-1 RAs to achieve substantial weight loss has increased, the need to preserve LBM has become a greater demand. It is also recognized that certain populations are at greater risk for loss of LBM during weight loss, including postmenopausal women, the elderly, and those with metabolic diseases [15,16,17]. Currently the best treatment available to slow or preserve LBM during weight loss is the implementation of high-protein diets and resistance exercise into the chosen weight loss regimen [15,16,17]. However, new pharmacologic methods are on the horizon as several promising drugs, which aim to prevent skeletal muscle loss during weight loss, are under development. If skeletal muscle is preserved while losing fat mass along with the benefits of weight loss, there would also be the benefits of improved metabolic parameters, better physiological glucose regulation, and sustained weight loss.

The purpose of this narrative review is to discuss key pharmacological treatments currently in phase II and III trials that show promising effects in promoting the preservation of skeletal muscle in the setting of weight loss and discuss their mechanisms.

## 2. Materials and Methods

With this narrative review, our goal is to provide a comprehensive review with structural formatting and thematic headings for pharmacological interventions that have shown promise in the preservation of muscle mass with weight loss, grounded in research. We chose to pursue a narrative review because this topic is gaining traction in research and thus, we wanted to include the variety of scientific evidence that is available at this time to provide a knowledge synthesis.

We queried PubMed, Medline, and Scopus for randomized controlled trials—phase II and phase III—with the following key words: obesity, weight loss, muscle loss, lean mass, and muscle preservation. We analyzed the studies conducted to date. We included commentaries and empirical articles regarding the above-mentioned studies to broaden the scope of perspective. We excluded animal studies.

The interpretation and analysis may have been biased towards pharmacological agents that have been researched more than the ones that have not. This is not to be inclusive of all research available on this topic.

Additionally, figures and tables were created using Illustrea (https://illustrea.com/).

## 3. Results

Skeletal muscle (SM) is a key component of the structure of the human body and supports the frame in more ways than physical shape. SM is important for metabolism, thermoregulation, and serves as a reservoir for amino acids. SM is an independent marker of metabolic health with functions encompassing glucose homeostasis (SM is responsible for 75% of all insulin-mediated glucose disposal), insulin sensitivity (insulin resistance at the level of SM has been shown to be a driver risk for type 2 diabetes), lipid oxidation (SM is a major site of aerobic metabolism), and basal metabolic rate (SM is a major contributor to resting energy expenditure) [15]. A 2018 study showed that low muscle mass was associated with a higher body fat percentage, but more importantly, an increased likelihood of metabolic disease, morbidity, and mortality [18]. SM is directly linked to quality of life and longevity; thus, preserving SM mass with ongoing weight loss is an important factor for overall health [12].

### 3.1. Signaling Mechanisms of Lean Body Mass Loss

LBM comprises all tissues expect for fat—muscle mass, bones, connective tissue, and skin. LBM loss is the result of a complex interplay between many signaling mechanisms. These mechanisms not only contribute to loss of LBM during weight loss regimens but also play a role in other conditions where LBM is lost, including sarcopenia, cachexia, metabolic disease, and muscle wasting diseases. Several key signaling components that lead to loss of LBM are discussed below. Table 1 provides a comprehensive list of these mechanisms.

#### 3.1.1. Insulin Signaling

Insulin signaling plays an important role in glucose disposal and skeletal muscle anabolism and maintenance. However, in the setting of decreased nutrient intake or insulin resistance, the muscle-protective effects of insulin signaling are lost. In patients participating in weight loss regimens—whether through caloric restriction, bariatric surgery, or pharmacologic methods—the perfect storm exists where fewer calories are being consumed, resulting in a decreased need for insulin release and, thus, lower insulin; combined with this is the potential insulin resistance seen in overweight and obesity where the response to insulin is lost, leading to decreased skeletal muscle preservation signaling.

Insulin elicits its effects by binding to the insulin/IGF (insulin-like growth factor) receptor in skeletal muscle, thus activating several downstream signaling pathways, including phosphatidylinositol-3 kinase (PI3K) and Akt (also known as Akt serine/threonine kinase), which modulate the mechanistic target of rapamycin complex 1 (mTORc1) [19,20]. A lack of insulin or insulin resistance prevents the activation of PI3K and Akt, and thus decreases mTORc1 signaling, which is a key anabolic signal in driving protein synthesis and cell growth through the activation of translational regulators [19,20]. Loss of insulin/PI3K/Akt/mTORc1 signaling is largely responsible for the loss of LBM seen with weight loss [19,20].

#### 3.1.2. AMPK Signaling

AMP-activated protein kinase (AMPK) is an energy-sensing protein in cells that is activated during low-energy states, such as during weight loss, when nutrient intake is low [21,22]. When AMPK is activated, it prevents mTORc1 activation by phosphorylating two of the regulatory proteins of mTORc1, Raptor and tuberous sclerosis complex 2 (TSC2) [21,22]. With AMPK-mediated phosphorylation of Raptor, part of the mTORc1 complex, mTORc1, is directly inhibited, preventing the anabolic and cell growth signaling that mTORc1 is responsible for driving [21,22]. AMPK also indirectly regulates mTORc1 by phosphorylating and activating TSC2 [21,22]. TSC2 is responsible for turning off the small GTP-ase, Rheb, another mTORc1 activator [21,22]. Thus, without mTORc1 signaling, the balance is shifted from anabolic protein synthesis to catabolic autophagy mechanisms, leading to loss of skeletal muscle. Additionally, AMPK signaling occurs alongside other atrophy pathways and they often converge.

#### 3.1.3. FoxO1/3 Signaling

Two pathways that are active in muscle atrophy and protein breakdown are the ubiquitin–proteosome and autophagy–lysosomal pathways [23]. The signaling of forkhead transcription factors 1 and 3 (FoxO1/3) is usually suppressed by Akt. However, when Akt activity is decreased in conditions of decreased insulin signaling, FoxO1/3 are upregulated, leading to the expression of E3 ligases Atrogin1 and MuRF1 (muscle ring finger 1), which target proteins for proteasomal degradation [23]. Additionally, when AMPK is activated, it differentially phosphorylates FoxO1/3, leading to the activation and downstream transcription of Atrogin1 and MuRF1, resulting in protein degradation [23]. Thus, the activation of FoxO1/3 leads to increased proteolysis and a net breakdown of skeletal muscle, leading to atrophy. FoxO1/3 signaling converges with insulin-Akt and AMPK signaling pathways.

#### 3.1.4. Myostatin Signaling

Myostatin, predominately expressed in skeletal muscle, is a negative regulator of muscle mass. Mutations in the myostatin gene lead to a hyper-muscular phenotype [24]. The negative regulatory effects of myostatin occur via signaling through the activin receptor type II (ActRIIB), leading to downstream signaling through SMAD2/3 (small mother against decapentaplegic 2/3), which promotes protein degradation, muscle loss, and atrophy [25]. The activation of SMAD2/3 leads to the transcription of atrophy-related genes such as Atrogin1 and MuRF1, similar to FoxO1/3 [25]. At the same time, myostatin decreases the transcription of genes leading to myogenesis, including MyoD (myoblast determination protein 1), MyoG (myogenin), MyHC (myosin heavy chain), and Pax7 [25]. Thus, the effects of myostatin are two-fold in that they increase atrophy while decreasing myogenesis; this is why this pathway has been the target for many drug developers aiming to preserve LBM.

Importantly, when initially transcribed, myostatin is released as a pro-myostatin [26]. Extracellular matrix proteases cleave the pro-myostatin, resulting in latent myostatin, which is also inactive and can be detected in serum. Following a final cleavage event, latent myostatin is transformed into a fully active mature myostatin which can bind to ActRIIB [24,26]. Various compounds have been developed to target the various forms of myostatin, which may lead to the preservation of LBM by the attenuation of protein degradation signaling and disinhibition of myogenic signaling [27].

#### 3.1.5. Cortisol Signaling

Cortisol binding to the glucocorticoid receptor (GR) in skeletal muscle leads to downstream disinhibition of the previously mentioned FoxO1/3, which can enter the nucleus and upregulate the transcription of E3 ligases Atrogin1 and MuRF1 (muscle ring finger 1), as well as several autophagy-related genes, leading to increased protein degradation [28]. By doing so, leucine and alanine can be released to be used by the liver for gluconeogenesis in conditions of low nutrients, such as during weight loss. Additionally, the caloric deficit is seen as a stressor, and the body responds by increasing the levels of circulating cortisol. The increase in cortisol signaling increases skeletal muscle protein breakdown, leading to skeletal muscle atrophy overtime.

#### 3.1.6. Autophagy Signaling

The autophagy–lysosomal protein degradation pathway has been noted to play a role in skeletal muscle atrophy in weight loss, aging, cachexia, disuse, inflammatory conditions, and chronic disease. Autophagy is mediated by numerous proteins, including LC3 (microtubule-associated protein 1 light chain 3), Beclin-1, and ULK1 (Unc-51-like kinase 1). These proteins play a role in shuttling proteins into the autophagosome as well as autophagosome lysosome fusion [29]. In the presence of AMPK signaling during weight loss, autophagy is upregulated; thus, there is breakdown of more proteins to release amino acids for gluconeogenesis, leading to loss of LBM overtime [29].

#### 3.1.7. Thyroid Hormone Signaling

Skeletal muscle is a major target for the thyroid hormone and expresses deiodinases to convert T4 into active T3. Once inside the cell, T3 can bind to proteins or the thyroid hormone receptor to elicit non-transcription- or transcription-related changes, respectively. The thyroid hormone upregulates proteins involved in the formation of slow twitch and oxidative muscle fibers by upregulating peroxisome proliferator-activated receptor coactivator 1α (PGC-1α) expression, which increases mitochondrial biogenesis [30]. Other myogenic signaling is also affected, leading to an imbalance between skeletal muscle generation and breakdown, producing muscle atrophy that presents as proximal muscle weakness [30].

#### 3.1.8. PGC-1α Signaling

PGC-1α (peroxisome proliferator-activated receptor gamma coactivator 1-alpha), as mentioned, is a regulator or mitochondrial function. PGC-1α controls transcription factors NRF-1 (Nuclear respiratory factor 1) and NRF-3 (Nuclear respiratory factor 3), which are responsible for increasing the expression of TFAM (Mitochondrial transcription factor A), ultimately leading to mitochondrial biogenesis, protecting from muscle atrophy [31]. Additionally, by suppressing FoxO3, PGC-1α prevents the transcription of Atrogin1 and MuRF-1, thus preventing muscle atrophy [31]. Under conditions of elevated AMPK activity, such as low energy states and insulin resistance, AMPK drives PGC-1α to be overexpressed [29,31]. This overexpression of PGC-1α leads to substantial mitochondria biogenesis, which causes mitochondrial uncoupling, leading to a significant decrease in ATP (Adenosine triphosphate) production [29,31]. Without ATP to support the muscle, atrophy increases and LBM is lost.

#### 3.1.9. TNF-α/IL-6 Signaling

Inflammation from various causes leads to increases in circulating inflammatory cytokines, including two major ones, tumor necrosis factor α (TNF-α) and interleukin 6 (IL-6). The expression of TNF-α and IL-6 are increased during chronic diseases such as diabetes [32]. Signaling through the TNF-α and IL-6 receptors converges on a downstream signaling factor, nuclear factor-κB (NF-κB), which acts as a nuclear transcription factor [29,32]. Once in the nucleus, NF-κB drives the transcription of genes of the ubiquitin–proteosome system, inflammatory mediators, and inhibitors of myogenesis [29,32]. Activation of the ubiquitin–proteosome system increases protein degradation; increased expression of inflammatory mediators causes increased infiltration of immune cells and myocyte death, increased protein degradation, and fibrosis and inhibition of myogenic signaling decreases muscle protein synthesis [32]. Overall, this leads to a phenotype of atrophy and LBM loss in a variety of conditions with increased proteolysis and decreased myogenesis.

#### 3.1.10. Leptin Signaling

Leptin is a signaling molecule released from adipose tissue, known as an adipokine. Under conditions of decreased nutrient intake or leptin resistance, a decrease in leptin signaling drives “starvation” signaling, leading to the increased breakdown of skeletal muscle proteins and, ultimately, skeletal muscle atrophy through decreases in energy expenditure [33]. Signaling through the leptin receptor in the hypothalamus increases JAK/STAT3 (Janus Kinase (JAK)/Signal Transducer and Activator of Transcription (STAT) 3) signaling and neuroendocrine regulation [33]. As a neuroendocrine molecule, decreases in leptin signaling can decrease various hypothalamus–pituitary signaling axes, which can also contribute to muscle atrophy. The effect on two of these hypothalamic–pituitary axes, thyroid and growth hormone/IGF-1, were discussed previously [33].

#### 3.1.11. Testosterone Signaling

Under conditions of low testosterone, the normal muscle preserving signaling via the androgen receptor decreases, leading to muscle atrophy. When testosterone is absent, there is a decrease in the protein anabolic signaling pathway PI3K-Akt-mTORc1 [34,35]. Additionally, testosterone inhibits myostatin signaling, so in its absence, there is more myostatin-driven proteolysis and thus increased muscle atrophy [34,35]. Overall testosterone is necessary to promote muscle growth and maintenance and prevent muscle atrophy.

### 3.2. Drugs Aimed at Preserving Lean Body Mass

With so many known mechanisms leading to skeletal muscle atrophy, there are a number of potential targets for preserving LBM. This has been an area of interest for some time, with a focus on skeletal muscle diseases like muscular dystrophy, cachexia, sarcopenia, disuse, and chronic disease. Recently, interest in these pharmacologic agents has increased with the increase in weight loss and subsequent LBM loss using GLP-1 RAs and GIP/GLP-1 RAs. The amount of total weight loss, and thus LBM loss, is consistently greater with GLP-1 RAs and GIP/GLP-1 RAs than with physiologic weight loss. As discussed, all forms of weight loss come with a reduction in not only fat mass, but in LBM as well. Thus, with the greater weight loss achieved with GLP-1 RAs and GIP/GLP-1 RAs comes increased concerns of the amount of LBM that is subsequently lost. This is exacerbated by the fact that the populations for which GLP-1 RAs and GIP/GLP-1 RAs are used to achieve weight loss in—namely, diabetic, obese, and/or elderly patients—potentially have lower amounts of LBM prior to weight loss; thus, preserving LBM in these populations is of great importance.

Several pharmaceutical companies have developed drugs that target muscle atrophy signaling to prevent the loss of LBM during weight loss. Some of these agents have been developed solely for use alongside GLP-1 RAs and GIP/GLP-1 Ras, with weight loss-induced loss of LBM as the focus, while others have been under development for various skeletal muscle diseases and are being investigated for a new use with the GLP-1 RAs and GIP/GLP-1 RAs. The overall goal of these novel agents is to prevent or lessen the loss of LBM. The following section discusses several classes of drugs based on the muscle atrophy signaling pathways they target.

#### 3.2.1. Drugs Targeting the Myostatin/Activin/ActRII Pathway

The drugs in this class use various mechanisms to target and prevent myostatin signaling with the goal of preserving lean body mass by inhibiting the catabolic effects of myostatin. Bimagrumab, trevogrumab, garetosmab, apitegromab, and landogrozumab are all promising, fully humanized monoclonal antibodies to various components of the myostatin–activin signaling pathway. RG6237 (also known as GYM329 or embrobart) is an antibody–antigen complex designed to bind latent myostatin, then, by binding the FcγRllb receptor, be internalized and degraded in a “sweeping” mechanism. Additionally, HS235 is a soluble receptor that binds and traps activin A.

By binding to activin type II receptors A and B (ActRIIA and ActRIIB), bimagrumab prevents binding of endogenous ligands myostatin, activin A, and growth differentiation factor 11 (GDF11), thus preventing activation of downstream Smad 2/3 muscle atrophy signaling. A phase II clinical trial with bimagrumab alone in patients with diabetes and a BMI between 28 and 40 showed positive outcomes [36]. Patients in the bimagrumab group had a decrease in total fat mass of 20.5% compared to just 0.5% in the placebo group, and all patients in the bimagrumab group lost at least 5% of total body fat mass [36]. The bimagrumab group also had a 3.6% increase in LBM compared to the placebo group, which had a −0.8% loss of LBM. The phase IIb BELIEVE trial looked at the effects of bimagrumab alone and in combination with semaglutide in adults with overweight or obesity [37]. When bimagrumab was used in combination with semaglutide, 92.8% of total weight loss was from fat mass compared to just 71.8% of weight loss being attributed to fat mass when semaglutide was used alone [37].

**Figure 1:**
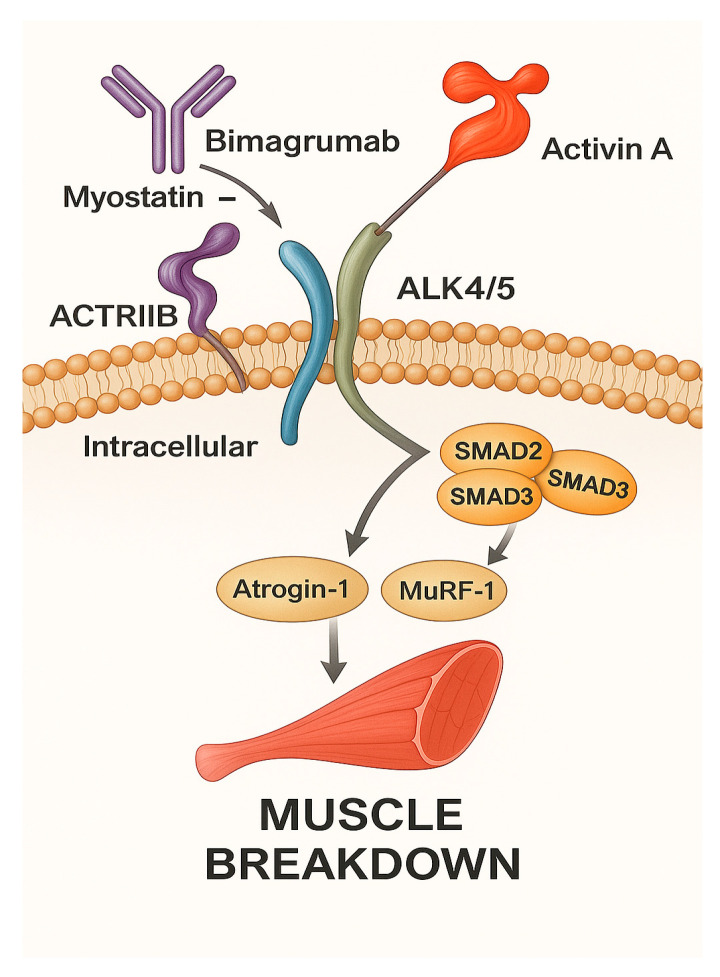
Pharmacological agents acting via the myostatin/activin/ActRII pathway.

Trevogrumab and garetosmab target myostatin and activin A, respectively. While myostatin is an established negative muscle regulator, in experiments in which its receptor ActRIIB was blocked, muscle growth was greater than myostatin blockade. This suggests the presence of other growth factors signaling through ActRIIB also working to negatively regulate skeletal muscle. This additional molecule was later found to be activin A [38]. Figure 1 below depicts the mechanism of action of pharmacological agents acting via the myostatin/activin/ActRII pathway.

In preclinical studies, trevogrumab was shown to be specific for both the pro- and latent forms of myostatin with little cross reactivity to GDF-11, which is the closest cousin to myostatin [39]. Garetosmab showed high affinity and specificity for activin A without cross reactivity as well [38]. Phase II clinical trials have been conducted administering trevogrumab alone or in combination with garetosmab in patients taking semaglutide for weight loss [40]. The phase II COURAGE trial showed that in the triplet group taking trevogrumab and garetosmab with semaglutide, there was a 27.1% decrease in fat mass compared to 15.7% with semaglutide alone and only a 2.0% change in LBM compared to 6.5% change in LBM with semaglutide alone [41]. These data show that by targeting both myostatin and activin A, greater weight loss can be achieved while preserving a greater percentage of LBM.

Similarly to others in this class, RG6237/GYM329 (emubrobart) is a promising latent myostatin-specific antibody engineered to include a novel sweeping function to continually clear myostatin from the plasma and muscle tissue while recycling the antibody [42]. Currently, a phase II clinical trial is underway to assess RG6237/GYM329 in combination with tirzepatide in patients with obesity or overweight with at least one weight-related comorbidity [43].

Apitegromab/SRK-015 is an anti-pro/anti-latent myostatin biologic being explored as a potential treatment for the preservation of LBM in obese or overweight patients taking GLP-1 RAs [44]. Apitegromab binds to the pro domain of myostatin and prevents extracellular proteolytic cleavage and subsequent activation of pro- and latent myostatin, thus preventing its binding to ActRIIB and subsequent downstream negative regulation of skeletal muscle [45]. The phase II randomized, double-blind, placebo-controlled, proof-of-concept trial EMBRAZE tested apitegromab for the preservation of LBM during weight loss with tirzepatide in non-diabetic patients with overweight or obesity [44,46]. EMBRAZE showed a significant increase in LBM compared to tirzepatide alone, with a difference of 1.9 kg of LBM [46]. There was a 15.8% greater mass loss due to fat in the apitegromab and tirzepatide group compared to the tirzepatide-only group, with no significant difference in total weight loss during the 24-week trial, indicating higher-quality weight loss when apitegromab is given with tirzepatide [46].

Landogrozumab is a monoclonal antibody that neutralizes myostatin. While there are not any trials looking at landogrozumab in overweight and obesity or in conjunction with GLP-1 RAs or GIP/GLP-1 RAs, landogrozumab has been looked at in other conditions of muscle atrophy, including sarcopenia and frailty. Patients who were 75 or older and had a fall in the past year who were randomized to landogrozumab showed a 0.43 kg increase in appendicular LBM [47]. In addition to the increases in LBM, they showed that these increases led to significant functional improvements, as assessed via stair climbing time, chair rise with arms, and gait speed [47]. These results indicate that landogrozumab may be beneficial in increasing functional LBM in other conditions besides sarcopenia.

With myostatin’s only known role being skeletal muscle atrophy, this pathway is a safe and effective target for the prevention of LBM loss with weight loss and other conditions of atrophy, with little risk of off-target effects. The promising results presented here show that the myostatin blockade can indeed preserve LBM in the setting of pharmacologic weight loss, which is of great importance and benefit to patients.

Drugs targeting myostatin are likely to continue to show benefits in preserving LBM during weight loss in future trials, which is of great interest for patients who experience substantial weight loss with GLP-1 RAs and GIP/GLP-1 RAs. Documented adverse events have been mild-to-moderate and include muscle spasms, diarrhea, nausea, and acne. These promising safety and tolerability profiles make them promising treatment options; however, these biologics are designed to be given as an IV infusion, thus having the downfall that patients go to a clinic for their treatment. Table 2 below shows the pharmacological agents in the class of the myostatin/activin/ActRII pathway, their downstream effects, and progress in research development, along with the area/subject they are being assessed for.

#### 3.2.2. Drugs Targeting Other Muscle Loss Signaling Pathways

Enobosarm (GTx-024) is a selective androgen receptor modulator (SARM) that selectively targets muscle [48]. Designed to mimic testosterone, enobosarm acts to promote skeletal muscle growth and maintenance by acting through PI3K-Akt-mTORc1 anabolic signaling [48]. In the phase IIb QUALITY clinical trial, enobosarm’s use was associated with a 71% reduction in LBM loss and a 27% increase in fat mass loss, when taken in combination with semaglutide [49]. In a maintenance extension of this phase IIb trial, it was seen that enobosarm use prevented more fat mass regain compared to the placebo [50]. With no serious adverse events reported and the most common mild adverse events being headache and back pain, enobosarm has a good safety profile [48]. Phase III clinical trials for cachexia and metastatic breast cancer have been performed but enobosarm has not yet received FDA approval. With its promising efficacy and safety, it is likely that enobosarm will continue to be researched for development in the arena of preservation of LBM loss during pharmacologic weight loss.

Tesamorelin (TH99507) is an FDA-approved growth hormone-releasing hormone (GHRH) analog. It promotes the release of growth hormone and leads to a subsequent increase in insulin-like growth factor 1 (IGF-1) [51]. Downstream IGF-1 signaling leads to increased activation of PI3K-Akt-mTORc1 anabolic signaling. Its approved indication is HIV (human immunodeficiency virus)-induced lipodystrophy, where it has been shown to reduce visceral and liver adipose tissue [51]. Tesamorelin has started being explored for use in overweight and obesity, insulin resistance, and nonalcoholic fatty liver disease [52]. It has been associated with an increase in abdominal muscle density and area along with a decrease in intramuscular and visceral adipose tissue [52]. Side effects are generally mild and include arthralgia, myalgia, and peripheral edema [51,52].

Anamorelin is a ghrelin agonist that is under phase III clinical investigation in patients with non-small cell lung cancer and cachexia. Ghrelin is known as the hunger hormone, as it is released from the empty stomach to reduce satiety signaling and promote feeding [53]. Once released, ghrelin binds to the growth hormone secretagogue receptor type 1a (GHS-R1a) in the hypothalamus, leading to the activation of NPY neurons, which promotes hunger [53]. In addition to promoting hunger, ghrelin signaling also promotes the release of growth hormone from the pituitary [53]. Once released, as previously discussed, growth hormone increases IGF-1 release via signaling through the insulin/IGF-1 pathway, promoting mTORc1 activation.

Phase III ROMANA 1 and 2 studies of anamorelin in patients with non-small cell lung cancer showed significant increases in LBM; however, muscle function did not improve, even with the increases in LBM [54]. Adverse side effects included hyperglycemia and diabetes [54]. While not yet approved by the FDA, anamorelin has been approved in Japan for use in improving cachexia. Anamorelin has not yet been explored for use in preserving LBM with weight loss.

Mecasermin (rhIGF-1) is a synthetic IGF-1 that signals through the IGF-1-mTORc1 pathway to promote LBM. Mecasermin was approved by the FDA in 2005 for the treatment of growth failure in children with IGF-1 deficiency and Rett Syndrome. Investigations showed that its use was associated with significant increases in LBM [55]. Adverse side effects of mecasermin include hypoglycemia, tissue overgrowth, and potential cancer. Investigations of mecasermin use with weight loss have not been performed; however, the risk of hypoglycemia may limit the use of mecasermin with GLP-1 RAs and GIP/GLP-1 RAs [55].

Tocilizumab is an FDA-approved monoclonal antibody that targets and blocks IL-6 receptor, which reduces downstream inflammatory signaling. As mentioned above, the presence of inflammatory signaling stops myogenic processes and activates muscle atrophy. Tocilizumab has been shown to increase LBM in trials for rheumatoid arthritis, where participants taking tocilizumab had increased appendicular LBM without changes noted in fat mass [56]. The increases seen in LBM points toward tocilizumab being a potential target agent to be used alongside GLP-1 RAs and GIP/GLP-1 RAs for preservation of LBM with weight loss; however, a trial has yet to be completed to investigate the potential outcome. Due to its inhibitory effect on inflammation, tocilizumab may be linked with an increased risk of infections. Potential for liver damage and changes in blood cell counts and cholesterol are all possible adverse effects as well.

Another potent target for reducing inflammation, which leads to muscle atrophy, is the blockade of tumor necrosis factor-alpha (TNF-α). Several TNF-α-blocking drugs have been approved by the FDA for various inflammatory and autoimmune conditions. These agents include etanercept, infliximab, adalimumab, and certolizumab. By blocking TNF-α-driven inflammatory signaling, myogenic signaling is preserved and muscle atrophy is diminished through decreased activation of NFκB. Following one year of treatment, patients with rheumatoid arthritis taking anti-TNF-α monoclonal antibodies showed increased total LBM, fat-free mass, and skeletal muscle mass with no changes in fat mass [57]. It was shown that the increases in LBM were associated with improved strength and walking [57]. While there have not been any direct studies performed combining anti-TNF-α agents with GLP-1 RAs or GIP/GLP-1 RAs, it has been shown that GLP-1 RAs alone seem to have some anti-inflammatory properties and they are associated with decreased levels of TNF-α [58]. This suggests that GLP-1 RAs or GIP/GLP-1 RAs and anti-TNF-α biologics may work synergistically together to induce weight loss and potentially spare LBM. Similarly to tocilizumab, other anti-TNF-α agents also have increased risk of infection, along with other side effects such as worsening of congestive heart failure and neurologic problems [59].

Table 3 below provides names, mechanisms, and the research progress for pharmacological agents targeting other muscle loss signaling pathways. Table 4 below lists key clinical trials that have been undertaken for the investigation of preservation of lean body mass with weight loss.

## 4. Discussion

This narrative review shows that most weight loss modalities, including calorically restrictive diets and pharmacological interventions, result in a significant loss of lean body mass. A large percentage of this consists of muscle mass, with the rest usually comprising supportive connective tissue. In addition, weight gain after weight loss usually does not restore the original percentage of lean mass, resulting in an overall higher body fat composition. There are several mechanisms that govern loss of lean body mass and, more specifically, muscle loss. These mechanisms have been exploited to develop several classes of agents to combat the loss of muscle during weight loss therapy. In addition, some agents, such as bimagrumab, may even augment weight loss when combined with GLP-1 agonists. However, most of these agents are still being investigated in research. Some of them may have concerning side effects such as muscle spasm and acne. Thus, more phase III trials are needed to confirm the benefits of these agents alone and also in combination with medications, such as GLP-1 RAs and GIP/GLP-1 RAs or dual agonists.

## Figures and Tables

**Table 1:** Signaling mechanisms driving lean body mass loss during significant weight loss.

Signaling Mechanism	Key Molecular Players	Effect on Muscle
↓ Insulin/IGF-1 → ↓ PI3K → ↓ Akt → ↓ mTORC1	Insulin, IGF-1, IRS-1, PI3K, Akt, mTORC1	↓ protein synthesis; ↑ autophagy
↑ AMPK activation → mTORC1 inhibition	AMPK, TSC2, Raptor	↓ protein synthesis; ↑ autophagy
↑ FoxO1/3 → ↑ Atrogin-1 & MuRF-1	FoxO1/3, Atrogin-1, MuRF-1	↑ targeted proteolysis
↑ Myostatin/Activin A → ActRIIB → SMAD2/3	Myostatin, Activin A, ActRIIB, ALK4/5, SMAD2/3/4	↓ myogenesis; ↑ atrophy gene transcription
↑ Cortisol → ↑ FoxO signaling	Cortisol, GR, FoxO	↑ proteolysis; ↑ amino-acid gluconeogenesis
↑ Autophagy–lysosome activation	ULK1, LC3, Beclin-1	↑ autophagy-driven protein degradation
↓ T3 & thyroid signaling	T3, thyroid receptor, PGC-1α	↓ protein synthesis; altered metabolism
↓ PGC-1α → ↓ mitochondrial biogenesis	PGC-1α, NRF1, TFAM	↓ oxidative capacity; ↑ AMPK activity
TNF-α / IL-6 → NF-κB activation	TNF-α, IL-6, NF-κB	↑ proteolysis; ↓ synthesis
↓ Leptin → ↓ anabolic neuroendocrine signaling	Leptin, JAK/STAT	↓ IGF-1, ↓ thyroid axis
↓ Testosterone → ↑ Myostatin, ↓ Akt → ↓ mTORC1, ↑ FoxO1/3 → ↑ Atrogin-1 & MuRF-1	PI3K, Akt, mTORC1, FoxO1/3, Atrogin-1, MuRF-1, Myostatin	↓ myogenesis; ↓ protein synthesis

**Table 2:** Pharmacological agents in the class of myostatin/activin/ActRII pathway, their downstream effects, progress in research development, and area of research study.

Drug	Mechanism	Phase/Area
Bimagrumab (BYM338)	ActRII blockade → inhibits myostatin/activin → ↓ SMAD2/3	Phase 2 (Obesity)
Trevogrumab (REGN1033)	Anti-myostatin mAb → ↓ SMAD2/3	Phase 2 (Obesity)
Garetosmab (REGN2477)	Anti-Activin A mAb → ↓ SMAD2/3	Phase 2 (Obesity)
RG6237/GYM329	Anti-latent-myostatin mAb → ↓ SMAD2/3	Phase 2 (Obesity adjunct)
Apitegromab/SRK-015	Anti-pro/anti-latent myostatin → ↓ SMAD2/3	Phase 2 (Weight-loss adjunct)
Landogrozumab (LY2495655)	Anti-myostatin mAb	Phase 2 (Sarcopenia/Frailty)

**Table 3:** Pharmacological agents targeting other muscle loss signaling pathways.

Drug	Mechanism	Phase/Area
Enobosarm (GTx-024)	Selective androgen receptor modulator → Akt/mTOR	Phase 2/3 (Sarcopenia/Frailty)
Tesamorelin (TH9507)	GHRH agonist → ↑ GH/IGF-1 → Akt/mTOR	Phase 3 (Body composition)
Anamorelin	Ghrelin agonist → ↑ GH/IGF-1	Phase 3 (Cancer cachexia)
Mecasermin (rhIGF-1)	IGF-1 receptor agonist → Akt/mTOR	Phase 3 (Metabolic/lean mass)
Tocilizumab	IL-6 receptor blockade → ↓ NF-κB catabolism	Phase 2 (Inflammatory muscle loss)
Anti-TNF agents	TNF-α blockade → ↓ NF-κB	Phase 3 (Inflammation-associated muscle loss)

**Table 4:** Key clinical trials for pharmacological agents that address preservation of lean body mass with weight loss.

Drug (Highest Phase)	Population & N	Primary Endpoint Result	Top 3 Adverse Effects (%)
Bimagrumab (Phase 2)	Obesity + T2DM; N = 75	Fat mass ↓ 20.5%; Lean mass ↑ 3.6%	Diarrhea 10–12%; Muscle spasms 7–9%; Nausea 5–6%
Trevogrumab (Phase 2)	Obesity (COURAGE); N ≈ 999	Lean mass preserved 50–80% vs. semaglutide	Muscle spasms 4.6–9.3%; Myalgia ≥10%; Infections ≥10%
Enobosarm (Phase 3)	Cancer cachexia; N = 159	Lean mass ↑ ~1.3 kg; Stair climb ↑ 18–22%	Nausea 23%; Anemia 17%; Constipation 17%
Tesamorelin (Phase 3)	HIV lipodystrophy; N = 404	Visceral fat ↓ 10.9%; IGF-1 ↑ 81%	Arthralgia 4%; Headache 3%; Edema 2–3%
Tocilizumab (Phase 3)	RA; N = 107	Lean mass ↑ 0.8–1.0 kg (*p* = 0.0097)	URI >10%; Nasopharyngitis 7%; ALT ↑ 6%

## Data Availability

No new data were created or analyzed in this study. Data sharing is not applicable to this article.

## Abbreviations

ActRIIA	Activin type II receptor A
ActRIIB	Activin receptor type II
ActRIIB	Activin type II receptor B
AMPK	AMP-activated protein kinase
ATP	Adenosine triphosphate production
CDC	Centers for Disease Control and Prevention
FDA	Food and Drug Administration
FFM	Fat-free mass
FoxO1/3	Forkhead transcription factors 1 and 3
GDF11	Growth differentiation factor 11
GHRH	Growth hormone-releasing hormone
GHS-R1a	Growth hormone secretagogue receptor type 1a
GIP RA	Glucose-dependent insulinotropic polypeptide receptor agonist
GIP-1	Glucagon-like peptide-1
GIP/GLP-1 RA or dual agonist	Dual glucagon-like peptide-1 receptor/glucose-dependent insulinotropic polypeptide receptor agonist
GLP	Glucose-dependent insulinotropic polypeptide
GLP-1 RAs	Glucagon-like peptide-1 receptor agonists
GR	Glucocorticoid receptor
HIV	Human immunodeficiency virus
IGF	Insulin-like growth factor
IL-6	Interleukin 6
JAK/STAT3	Janus Kinase (JAK)/Signal Transducer and Activator of Transcription (STAT) 3
LBM	Lean body mass
LC3	Microtubule-associated protein 1 light chain 3
mTORc1	Mechanistic target of rapamycin complex 1
MuRF1	Muscle ring finger 1
MyHC	Myosin heavy chain
MyoD	Myoblast determination protein 1
MyoG	Myogenin
NFκB	Nuclear Factor kappa-light-chain-enhancer of activated B cells
NPY	Neuropeptide Y
NRF-1	Nuclear respiratory factor 1
NRF-3	Nuclear respiratory factor 3
Pax7	Paired box gene 7
PGC-1α	Peroxisome proliferator-activated receptor gamma coactivator 1-alpha
PI3K	Phosphatidylinositol-3 kinase
SARM	Selective androgen receptor modulator
SM	Skeletal muscle
SMAD2/3	Small mother against decapentaplegic 2/3
T2DM	Type 2 diabetes mellitus
TFAM	Mitochondrial transcription factor A
TNF-α	Tumor necrosis factor alpha
TSC2	Tuberous sclerosis complex 2
U.S.	United States
ULK1	Unc-51-like kinase 1
